# Target-Responsive DNA Hydrogels with Encapsulation and Release Properties Using Programmable CRISPR-Cas12a

**DOI:** 10.1021/acschembio.5c00355

**Published:** 2025-07-09

**Authors:** Ram J. Tharu, Emmett Hanson, Mehmet V. Yigit

**Affiliations:** † Department of Chemistry, University at Albany, State University of New York, 1400 Washington Avenue, Albany, New York 12222, United States; ‡ The RNA Institute, University at Albany, State University of New York, 1400 Washington Avenue, Albany, New York 12222, United States

## Abstract

We report the development of a DNA hydrogel that disassembles and releases its payload in response to a target of interest. The DNA hydrogel is assembled from Y-shaped DNA motifs with polyA domains and cross-linked *via* the small molecule cyanuric acid through hydrogen bonding. The hydrogel’s structural integrity was rapidly assessed using a simple, instrumentation-free capillary migration assay that provides results within seconds. To evaluate its responsiveness to enzymatic degradation, the hydrogel was exposed to nonspecific nuclease activity using *DNase I*, resulting in increased mobility and decrease in fluorescence. Later, CRISPR-Cas12a was incorporated to enable programmable, target-specific hydrogel disassembly using a conserved genomic region from . Guided by crRNA sequences, the target sequences activated Cas12a to selectively degrade hydrogels. This process enabled the controlled release of various payloads, including a small-molecule drug, a fluorescent dye, a nanoparticle-based MRI contrast agent conjugated to a chemotherapeutic agent, and a model protein. To evaluate whether the hydrogel disassembly can be selectively programmed to an intended target, we tested its responsiveness against two serotypes of *Salmonella*, *i.e.*, conserved genomic regions from and . To test the disassembly of this novel DNA hydrogel in the presence of a full genome, we tested the hydrogel with the genome. The target genome induced an increase in the hydrogel’s mobility and loss in fluorescence with as few as 50 copies of full genome. The results demonstrate the potential of these CRISPR-responsive DNA hydrogels as intelligent platforms for target-induced imaging and therapeutic agent release, and biosensing applications.

## Graphical abstract

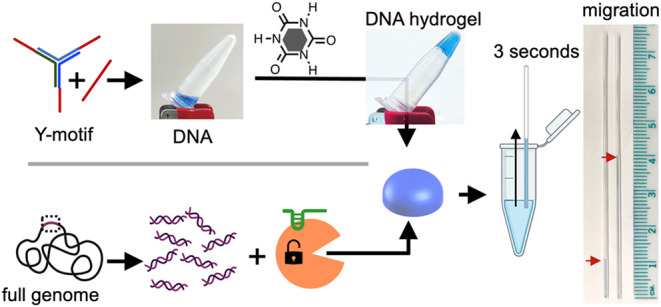

## Introduction

Deoxyribonucleic acid (DNA) is the fundamental genetic material that encodes biological information. Beyond its genetic role, DNA is a versatile biopolymer capable of folding into well-defined nano- and microstructures.^1–3^ Its inherent programmability, structural predictability, and self-assembly behavior make DNA a multifunctional material for diverse applications, including nanofabrication, biosensing, and drug delivery.^4–6^ Among emerging DNA-based materials, DNA hydrogels have attracted growing interest.^7,8^ While still in the early stages of development, hydrogels offer promise in the fields of targeted therapeutics and responsive biosensing platforms due to their biocompatibility and low immunogenicity properties.^9–13^

Traditionally, DNA hydrogels have been fabricated by attaching DNA strands to synthetic polymers.^14,15^ In a 2016 report, Sleiman and co-workers demonstrated that the small organic molecule cyanuric acid (CA) can self-assemble polyadenylated nucleic acids *via* hydrogen bonding, due to its three thymine-like faces to bind Adenine base.^16^ We have used this approach to form hydrogels composed solely of DNA, capable of encapsulating small molecules, nanoparticles, and proteins within their matrix, by employing branched polyadenylated Y-motif DNA structures that self-assemble with CA.^17^ Here, we demonstrate that these hydrogels can respond to specific genomic DNA markers, undergoing disassembly and payload release using the CRISPR-Cas12a complex.

The CRISPR-Cas12a system, derived from clustered regularly interspaced short palindromic repeats (CRISPR), functions as a programmable nuclease complex guided by a CRISPR RNA (crRNA).^18,19^ Upon recognition and binding of a specific double-stranded DNA (dsDNA) target that is complementary to its crRNA, Cas12a becomes activated and initiates indiscriminate cleavage of nearby DNAs.^20,21^ This collateral cleavage activity is a distinctive feature that has enabled the development of highly adaptable CRISPR-based diagnostic tools.^22–24^ The system’s target specificity can be readily tuned by simply modifying the crRNA sequence, making it a flexible platform for molecular recognition. In this study, we utilized the programmability of the CRISPR-Cas12a system to induce the disassembly of DNA hydrogels in response to the conserved genomic regions of .

*Salmonella* is a leading cause of foodborne illness worldwide, presenting serious risks to both public health and food safety.^25^ This Gram-negative bacterium is known for its capacity to contaminate a wide variety of food products. In this study, we used conserved genomic regions of as model systems to trigger the disassembly of DNA hydrogels in a target-specific manner.^22,23^ To evaluate the specificity of the system, a highly specific genomic region from was used.

## Results and Discussion

We first investigated whether a DNA hydrogel could be formed by using the Y-motif in combination with cyanuric acid (CA) and the supporting polyA strands. CA has three thymine-like faces,^16,26^ which enable the assembly of polyA strands into a DNA network. First, the Y-motif was constructed from three equimolar single-stranded DNAs that spontaneously hybridize to form a three-armed, double-stranded structure (Figure 1a).^27^ To enable interaction with CA, a single-stranded polyA domain (A15) was added to the 5′ end of each strand (represented by the red line on strands i, ii, and iii in Figure 1a).^17^ Upon hybridization, these polyA sequences extend outward from the core of the Y-motif, providing an assembly point for the hydrogel.

**Figure 1:**
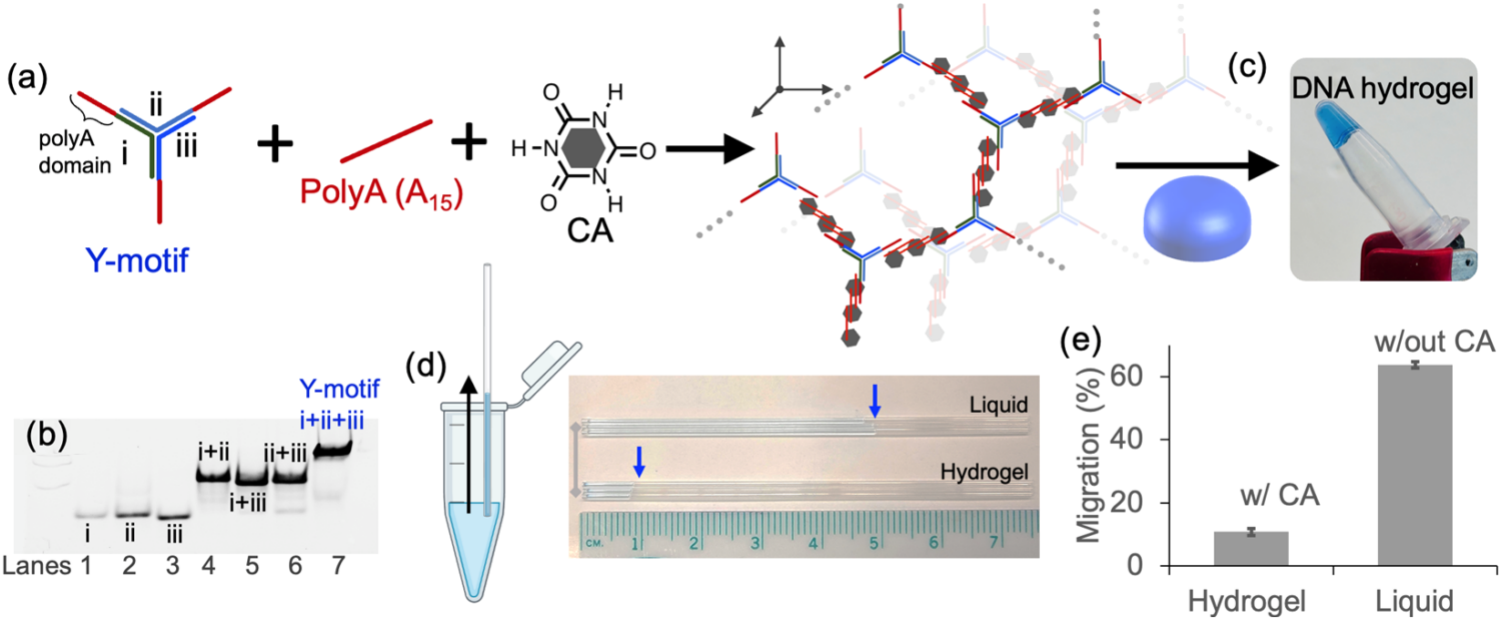
(a) Small-molecule cyanuric acid (CA) facilitates the assembly of a three-armed, Y-shaped DNA motif and unmodified polyA (A15) strands into (c) as a DNA hydrogel stained with Bromophenol Blue. (b) Gel electrophoresis confirms the stepwise formation of the Y-shaped DNA motif from one, two, and three DNA strands. (d, e) Capillary images and measured migration showed that the DNA hydrogel (with CA) exhibits reduced migration compared to the DNA mixture without CA (in liquid form). Experiments were performed in triplicate.

To validate the Y-motif assembly, the three individual DNA strands were first analyzed by gel electrophoresis. Lanes 1, 2, and 3 (Figure 1b) show distinct bands for each component. Pairwise hybridizations of the strands (i + ii, i + iii, and ii + iii) were then tested in a 1:1 ratio, yielding single bands with slower mobility, consistent with dimer formation (lanes 4–6). Finally, all three strands were combined in equimolar amounts (i + ii + iii) to form the complete Y-motif.^27^ Across all seven combinations, the fully assembled Y-motif showed a single, slowly migrating band, indicating successful hybridization and triplex complex formation (lane 7, Figure 1b).

We later tested whether this three-armed motif provides branching for DNA network formation in the presence of CA. Additional free A15 strands, at a 5:1 ratio relative to the Y-motif, were included in the mixture to further promote spacing, branching, and cross-linking within the DNA network. To aid the visualization of the hydrogel, Bromophenol Blue was used as a staining dye (Figure 1c). The resulting mixture formed a DNA hydrogel that retained its structural integrity when inverted and gently shaken.

After observing hydrogel formation, we performed a migration assay to define a quantitative parameter that is quick, cost-efficient, and instrument-free.^28,29^ We investigated whether the resulting hydrogel exhibited reduced migration, which could later be used to study its response to enzymatic degradation. To assess this, we conducted a capillary-based migration assay using DNA mixtures containing the Y-motif, polyA strands, and Bromophenol Blue dye, both in the presence and absence of cyanuric acid (CA). In this setup, the hydrogel was prepared in a 1.5 mL microtube.^28^ A 7.5-cm-long capillary tube (0.4 mm inner diameter) was vertically inserted into the DNA solution for 3 s and then removed (Figure 1d). The migration of the mixture was determined by measuring the extent to which the DNA sample traveled within the capillary.

Images of the capillaries revealed that samples containing CA, which formed a hydrogel, exhibited significantly slower migration compared to those without CA (Figure 1d). This reduction in distance traveled in the capillary was further quantified, confirming that CA-induced hydrogel formation reduced the movement of the DNA mixture (Figure 1e). After establishing that migration in the capillary can distinguish DNA in hydrogel form from the DNA mixture in liquid state, we next examined whether the hydrogel could be degraded by a nuclease. Our approach first focused on demonstrating hydrogel degradation through fluorescence using *DNase I*, followed by correlating this nonspecific degradation with changes in migration.

Deoxyribonuclease I (*DNase I*) is an endonuclease enzyme that cleaves the DNA phosphodiester bonds.^30^ By treating the hydrogel with *DNase I*, the phosphodiester bonds in the Y-motif would break and disassemble, no longer maintaining a hydrogel property. The *DNase I* activity can be monitored by using the PicoGreen fluorescence assay. PicoGreen is a fluorescent dye (Ex: 480 nm, Em: 525 nm) that selectively binds to double-stranded DNA (dsDNA), generating a strong fluorescence signal upon binding.^31,32^ As the DNA within the hydrogel is degraded by *DNase I*, the fluorescence intensity of PicoGreen is expected to decrease, indicating hydrogel disassembly (Figure 2a). This degradation can be further tested by a migration assay conducted in capillary tubes.

**Figure 2:**
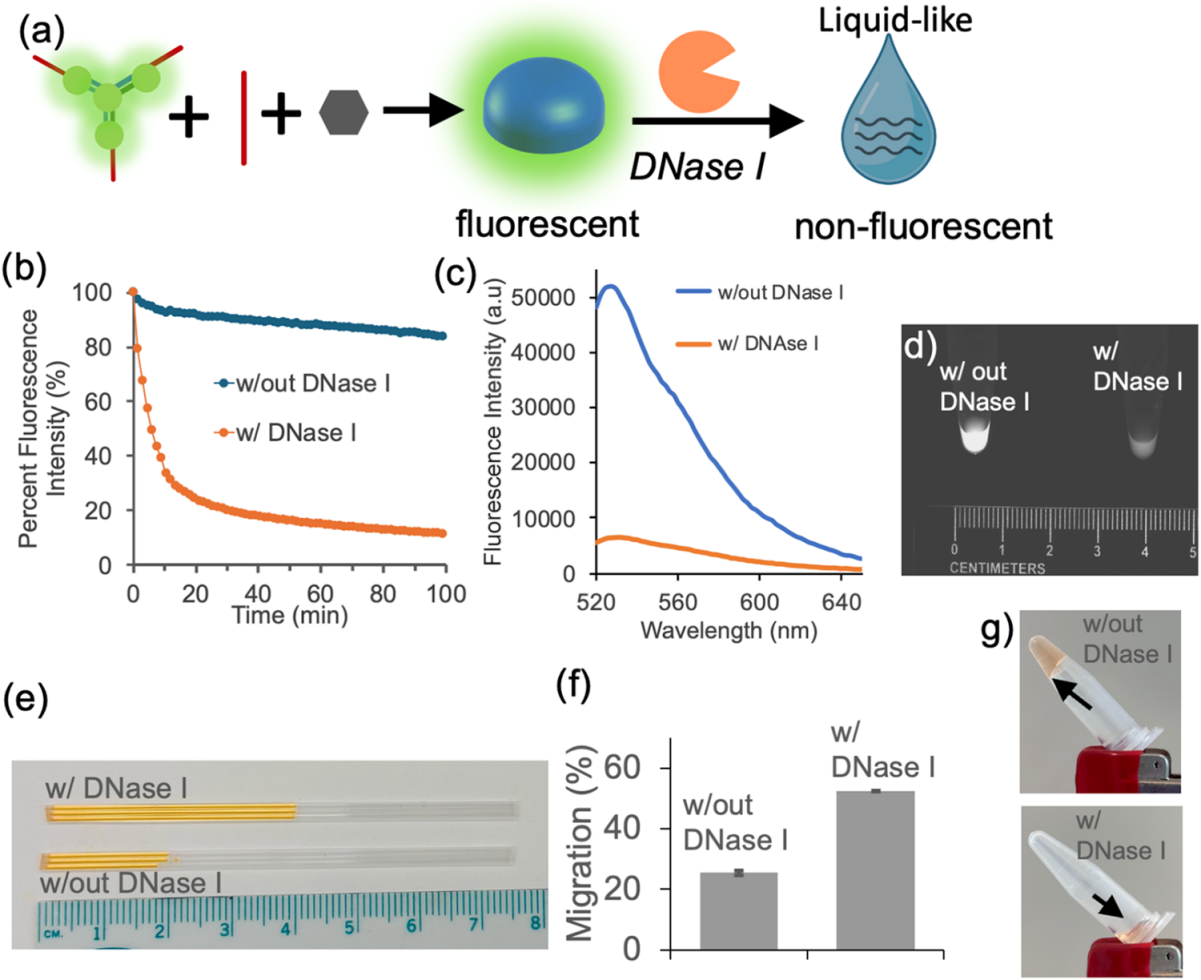
(a) Schematic illustration of *DNase I*-induced degradation of the DNA hydrogel. (b–d) Fluorescent readings from the DNA hydrogel stained with PicoGreen, a dsDNA-specific fluorescent dye, show a loss of fluorescence upon *DNase I* treatment, confirming degradation of the double-stranded DNA network. (d) Fluorescence image of the hydrogel in the microtube shows reduced fluorescence following degradation. The ruler is provided as a reference to illustrate the dimensions of the tube. (e, f) Increased mobility of the DNA hydrogel following *DNase I*-induced degradation, demonstrated through a capillary-based migration assay. The capillaries were inserted into the hydrogel solution for 3 s, and the images were quantified immediately afterward. Shown are representative (e) capillary images, (f) quantification of migration, and (g) photos of hydrogel samples in microtubes with and without *DNase I* treatment. Experiments were performed in triplicate.

As shown in Figure 2b–c, *DNase I* treatment led to a marked reduction in fluorescence intensity, monitored both over time and by end-point spectra at ∼525 nm. Semiquantitative fluorescence imaging of the hydrogels with and without *DNase I* further supported this spectroscopic observation (Figure 2d). Following confirmation of DNA cleavage through fluorescence assay, we assessed whether the degradation could also be detected through changes in capillary-based migration. As shown in Figure 2e,f, *DNase I*-treated hydrogels exhibited increased migration, supported by both representative capillary images and quantitative analysis. To visually evaluate the structural integrity of the hydrogels, the microtubes were inverted and are visualized in Figure 2g. Intact hydrogels remained at the bottom of the inverted tube despite gentle shaking, while *DNase I*-treated samples lost structural cohesion and flowed downward, suggesting the loss of gel-like properties.

After confirming that nuclease treatment can degrade the hydrogel structure and affect its mobility, we investigated whether this behavior could occur in a target-specific manner. We specifically investigated whether detection of a highly specific model pathogenic genome region could trigger DNA hydrogel degradation and subsequently affect its mobility. CRISPR-Cas12a, a nuclease activated by binding to a specific DNA target *via* its crRNA, was used for this purpose. Upon recognition of the double-stranded DNA (dsDNA) target, the Cas12a-crRNA complex becomes catalytically active for the cleavage of the DNA-based hydrogel (Figure 3a).

**Figure 3:**
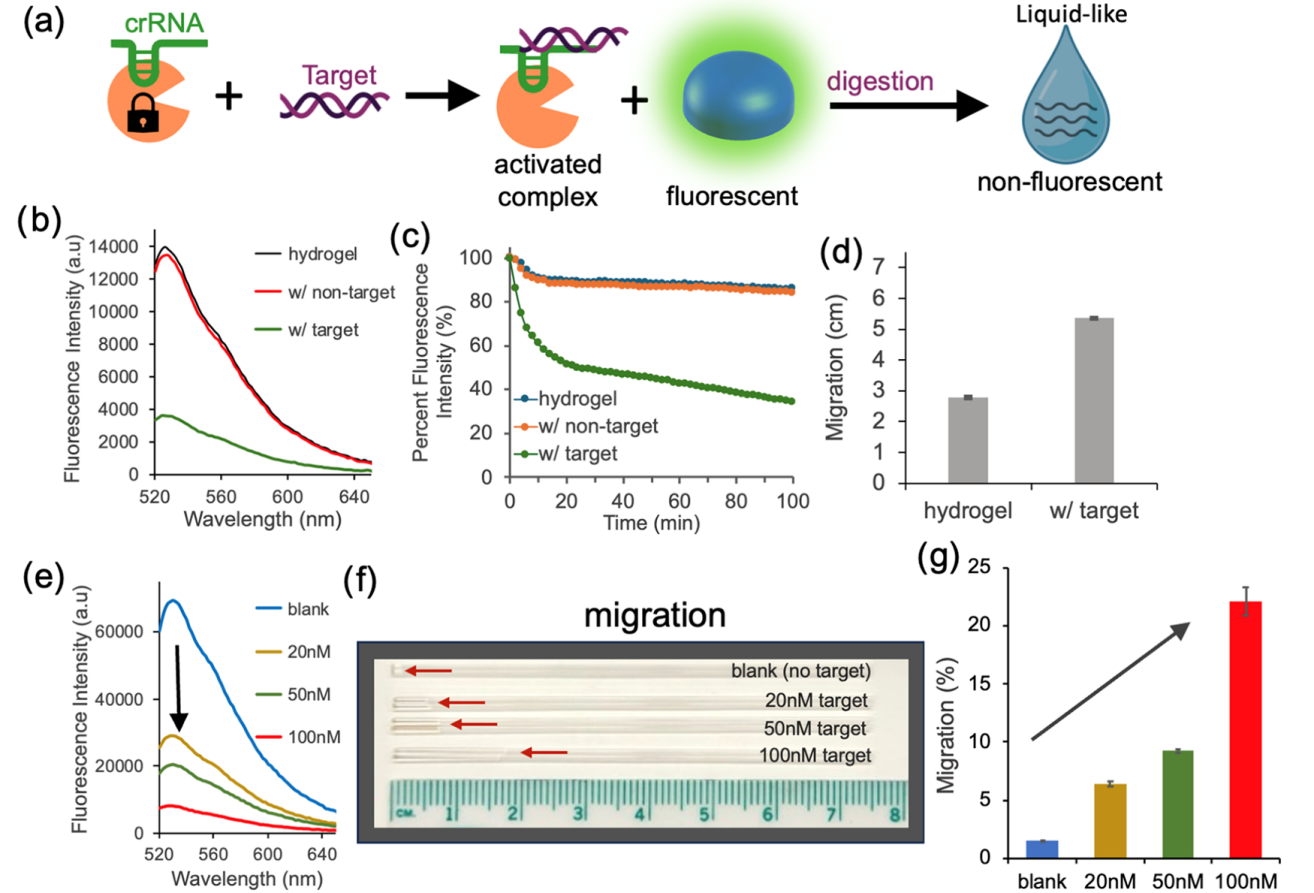
(a) Schematic illustration of the Cas12a-crRNA complex programmed with a specific genomic target to induce degradation of the DNA hydrogel. (b, c) Fluorescence measurements of PicoGreen-stained DNA hydrogels showing a decrease in fluorescence upon target recognition. (d) Capillary-based migration assay demonstrating increased hydrogel mobility following target-induced degradation. (e) Concentration-dependent response was observed, where increasing target DNA concentrations led to reduced fluorescence intensity, which correlated with (f) capillary images and (g) quantitative analysis of migration in the capillary. Experiments were performed in triplicate.

The CRISPR-Cas12a system, known for its high specificity, has been widely applied in nucleic acid-based detection.^33^ In this system, the Cas12a enzyme is guided by a CRISPR RNA (crRNA) complementary to the target dsDNA.^34^ Recognition of the target is essential to activate Cas12a’s trans-cleavage activity (Figure 3a).^22–24,35^ We utilized the CRISPR-Cas12a system to induce degradation of the DNA hydrogel. Like the *DNase I* assay, PicoGreen was intercalated into the DNA hydrogel matrix to enable the fluorescence-based monitoring of DNA degradation. We employed a conserved genomic region of (tDNA_Typh3_ + cDNA_Typh3_) to activate the Cas12a-crRNA complex. Upon target recognition, a gradual decrease in fluorescence at 525 nm of PicoGreen was observed, as confirmed by end-point spectra and intensity measurements over a 100 min period (Figure 3b,c).

The cleavage kinetics is observed to slow as substrate concentration decreases (Figure 3b), a behavior commonly observed in Cas12a systems, particularly when the substrate is a particulate material such as a hydrogel. Given that Cas12a activity is strongly influenced by the binding efficiency between crRNA and the target, a relatively high target concentration (100 nM) was used to ensure efficient activation and to accelerate the reaction in this setup with high cleavable hydrogel DNA content.

Nevertheless, this decrease in fluorescence was not observed when the Cas12a-crRNA complex was exposed to either no target or a nonspecific dsDNA from a different serotype, , (tDNA_Ente_ + cDNA_Ente_) that does not activate the complex. The migration assay further demonstrated that hydrogels treated with both the Cas12a-crRNA complex and the specific target DNA exhibited increased mobility compared to those treated with Cas12a-crRNA and nontarget DNA (Figure 3d).

To investigate whether this response could be concentration-dependent, we examined the hydrogel mobility using smaller sample volumes (50 μL) and varying target concentrations (0, 20, 50, and 100 nM). We observed a concentration-dependent decrease in fluorescence (Figure 3e) and a corresponding increase in capillary-based migration, confirming the tunability of the system (Figure 3f,g). Prior to this study, the optimal concentration of Cas12a was determined by testing various concentrations of the Cas12a-crRNA complex using a fixed concentration of the target (Figure S1).

To evaluate whether the hydrogel disassembly can be selectively programmed to the intended target, we tested its responsiveness against two serotypes of *Salmonella*, *i.e.*, conserved genomic regions from and . When Cas12a-crRNA was designed to recognize a conserved genomic region of , the hydrogel exhibits a shift in migration exclusively in the presence of , with no response to (Figure 4a). Conversely, when programmed for , a migration change is observed only in the presence of , but not with (Figure 4b). The results demonstrate that the hydrogel can be programmed to a target of interest by solely changing the crRNA of the Cas12a-crRNA complex.

**Figure 4:**
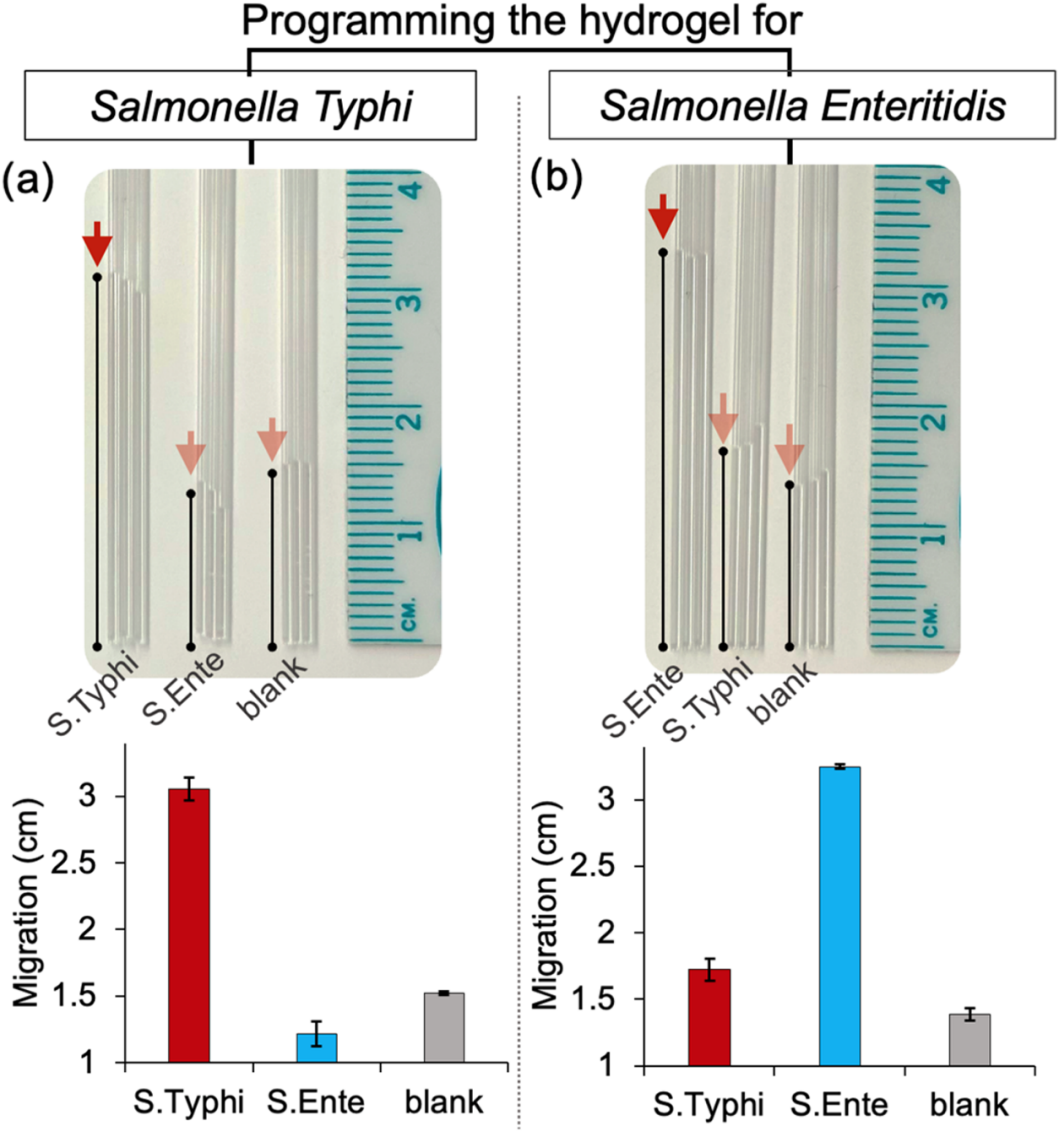
Programming the CRISPR-responsive DNA hydrogel for and , separately. Capillary-based migration changes are observed when the Cas12a-crRNA complex is programmed for (a) Salmonella typhi but not , or (b) but not . Experiments were performed in triplicate.

After confirming that the hydrogel undergoes structural degradation in response to the target of interest, we further investigated whether hydrogels encapsulating various payloads, *i.e.*, small molecules, nanoparticles, or proteins, could exhibit similar target-responsive release behavior. To facilitate monitoring, we used payloads with intrinsic fluorescence properties or labeled with fluorescent markers. The payloads used in this study were tetramethylrhodamine (TAMRA; Ex: 555 nm, Em: 580 nm), a small fluorescent dye widely used in biological studies; doxorubicin (Dox; Ex: 500 nm, Em: 590 nm), a chemotherapeutic small molecule with inherent fluorescence; dextran-coated iron oxide nanoparticles labeled with either doxorubicin (MN_Dox_) or cy5.5 (MN_cy5.5_; Ex: 675 nm, Em: 695 nm); and Red Avidin, a fluorescently labeled form of avidin (Ex: 590 nm, Em: 615 nm), Table S2. The iron oxide nanoparticles (MN) used in this study function as T2-weighted MRI contrast agents studied in clinical prostate cancer imaging,^36^ while cy5.5 was selected for its far-red fluorescence and high photostability, making it well suited for *in vivo* optical imaging applications.^37,38^

Initially, the payloads were successfully encapsulated within the hydrogel, which was sufficiently stiff to maintain its structural integrity (Figure 5a). Bright-field and fluorescence images confirmed the uniform distribution and encapsulation of the payloads within the hydrogel matrix. To evaluate the hydrogel’s responsiveness to the intended target and its facile programmability, we conducted a fluorescent capillary mobility assay using 20 nM target (tDNA_Typh5_ + cDNA_Typh5_) which is determined from a different conserved region of the and targeted by a crRNA specifically designed for this region, Table S1. In all tested cases, the hydrogels exhibited an increase in migration compared to untreated controls, indicating a target-induced structural change (Figure 5b,c). The vertical lines in Figure 5b represent the fluorescence images of the hydrogel that traveled through the capillary tubes. These results demonstrate that the hydrogel not only responds specifically to the target by altering its integrity but also effectively encapsulates a variety of payloads that can be unloaded upon target-triggered degradation of the DNA hydrogel.

**Figure 5:**
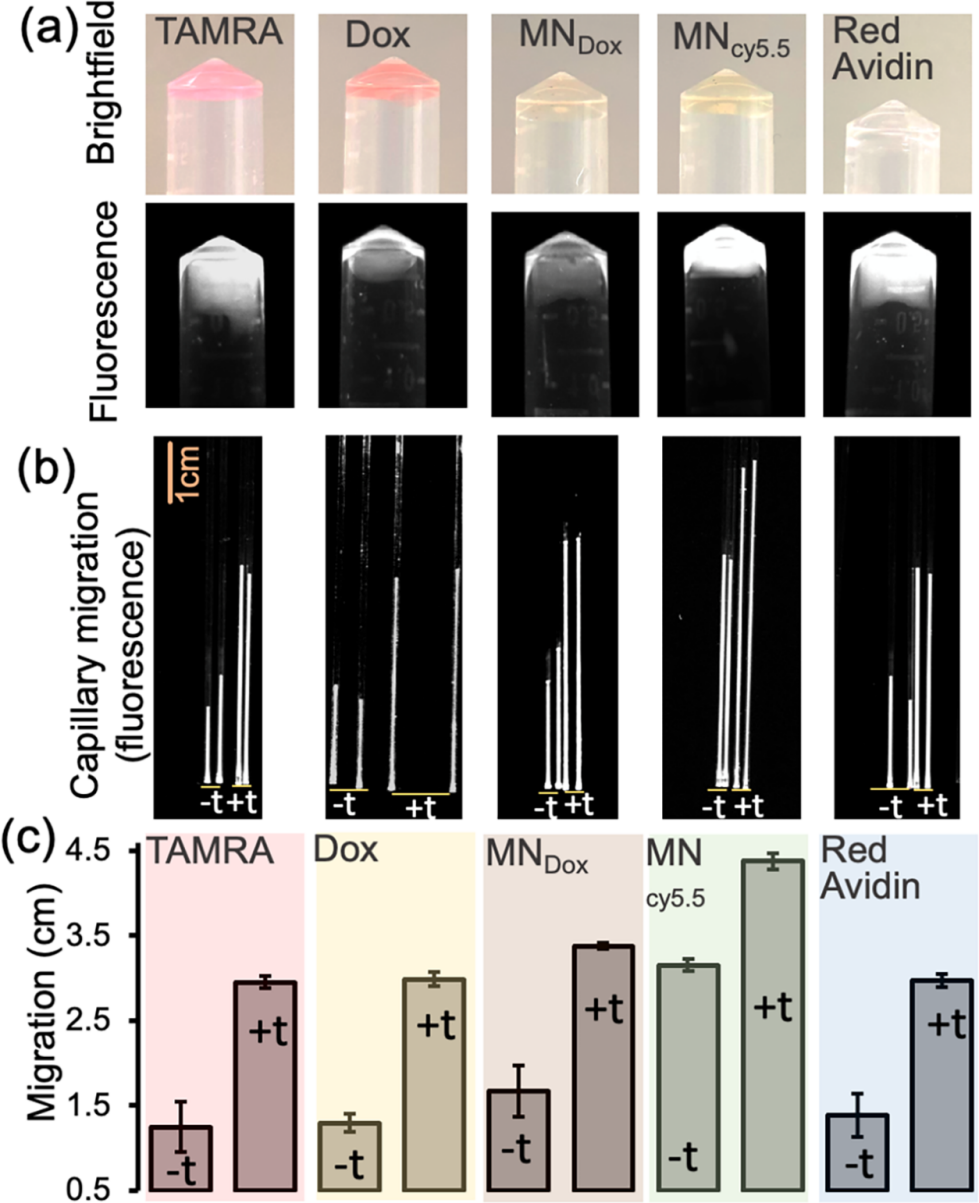
(a) Bright-field and fluorescence images of DNA hydrogels encapsulating various payloads: TAMRA, Doxorubicin (Dox), magnetic nanoparticle-conjugated to Dox (MN_Dox_), magnetic nanoparticle-conjugated to Cy5.5 (MN_cy5.5_), and Red Avidin, respectively. (b) Upon detection of a model genomic fragment from (S.Ty.5), increased hydrogel migration was recorded by fluorescence imaging in capillary tubes. The fluorescence images of the hydrogel moved into the capillaries were captured by using a Bio-Rad imaging system. The vertical lines labeled “+t” and “–t” represent hydrogels treated with and without the target, respectively. (c) This response was quantified by measuring the distance the hydrogel traveled within the capillary tube with (+t) and without (−t) target. Experiments were performed in duplicate.

Finally, to demonstrate that detection can be achieved using the full genome rather than synthetic amplicons, PicoGreen-stained hydrogels were tested with the complete genome. An isothermal amplification reaction (RPA)^39^ was first performed to generate amplicons (dsDNA_Typh3_) from the whole genome, followed by a CRISPR-Cas12a reaction (Figure 6a). As expected, the fluorescence intensity of PicoGreen decreased in samples containing as few as 50 copies of the full genome compared to control samples treated identically but lacking the target genome (Figure 6b). Additionally, the capillary migration assay revealed enhanced migration in hydrogels exposed to the 50 copies of the full genome (Figure 6c). These results confirm the platform’s capability to detect low amounts of full genomic DNA through both fluorescence and mechanical response. Compared to our earlier CRISPR-Cas12a studies using fluorescent silver nanoclusters, hybridization chain reaction, and a Toehold RNA switch, where we detected as few as 33, 40, and 100 copies of the full pathogenic genomes, respectively,^22–24^ observing a migration change with 50 copies is reasonable.

**Figure 6:**
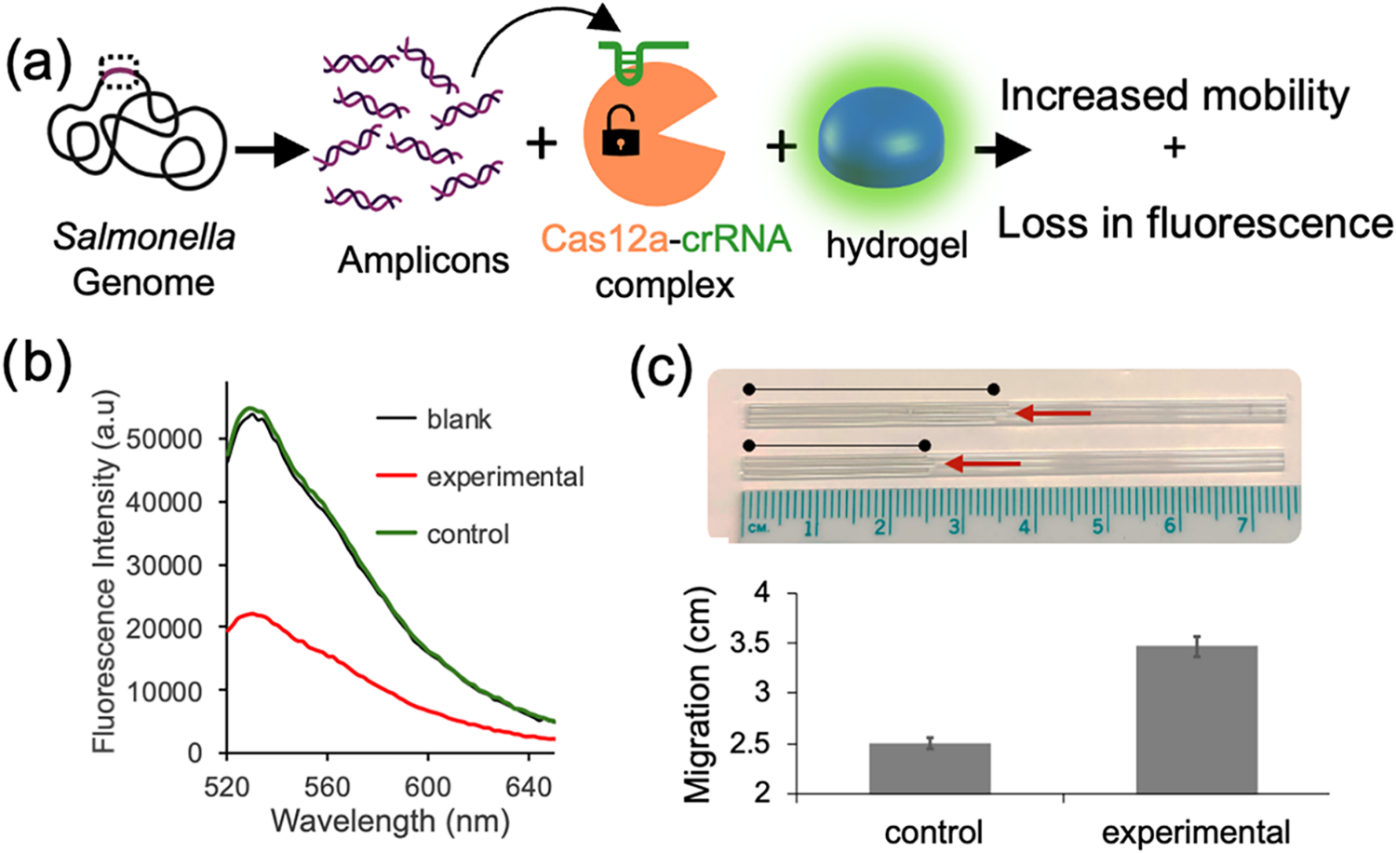
(a) Schematic illustration of the full-genome-triggered chain reaction leading to degradation of the DNA hydrogel.  (b) Fluorescence measurements of PicoGreen-stained DNA hydrogels show a decrease in signal upon recognition of the target genome.  (c) Capillary-based migration assay indicated increased hydrogel mobility following target-induced degradation. Experiments were performed in triplicate.

## Conclusions

In this study, we developed a DNA hydrogel primarily composed of Y-shaped DNA motifs with polyA domains, assembled into a hydrogel structure through the addition of a small-molecule cross-linker. The stiffness and integrity of the hydrogel were rapidly assessed using a simple capillary mobility assay, which provided results in a few seconds. We first evaluated the hydrogel’s response to nonspecific nuclease activity using *DNase I*, which resulted in increased mobility of the hydrogel following degradation. This degradation was further validated using a PicoGreen fluorescence assay, which showed a strong correlation with the migration data. To introduce target-specific degradation rather than nonspecific degradation, we employed the CRISPR-Cas12a system to degrade the DNA hydrogel in the presence of a target dsDNA. Given that the hydrogel is primarily composed of DNA, trans-cleavage sites for Cas12a are highly accessible and abundant. Guided by crRNA sequences complementary to specific genomic regions from , Cas12a enabled programmable hydrogel degradation and the controlled release of various payloads. To assess the disassembly of this DNA hydrogel in response to a complete genome, we tested its behavior with the genome. The presence of as few as 50 copies of the full genome led to an increased hydrogel mobility and a noticeable reduction in fluorescence. This investigation is relevant to the field of chemical biology, as a small chemical molecule was used to modulate the stiffness of the DNA motifs, enabling hydrogel formation. While further development is required for precise, target-specific release of therapeutic and imaging agents, our results represent a promising step toward the design of DNA-based hydrogels for applications in biomedical research and diagnostics.

## Supplementary Material



## Notes

Experimental details, list of materials, oligonucleotide sequences (Table S1), list of payloads (Table S2), and additional data (Figure S1) (PDF)
