# Supplementary material for: Target-Responsive DNA Hydrogels with Encapsulation and Release Properties Using Programmable CRISPR-Cas12a
Source: ACS Chem Biol. 2025 Jul 9;20(7):1805–12. doi: 10.1021/acschembio.5c00355 (PMC12281480; doi:10.1021/acschembio.5c00355)
Supplement: Supplementary file 1 [file cb5c00355_si_001.pdf]

Supporting Information

Target-responsive DNA hydrogels with encapsulation and release properties using programmable CRISPR-Cas12a

Ram J. Tharu[†], Emmett Hanson[†], and Mehmet V. Yigit[*,†,‡]

† Department of Chemistry,
University at Albany, State University of New York,
1400 Washington Avenue, Albany, New York 12222, United States.

‡ The RNA Institute,
University at Albany, State University of New York,
1400 Washington Avenue, Albany, New York 12222, United States.

*Correspondence:

Tel: (1) 518-442-3002

myigit@albany.edu

Keywords: DNA hydrogel • fluorescence • CRISPR • sensor • genome

Experimental Section.

Materials and Methods. Cyanuric acid ($C_3H_3N_3O_3$) was purchased from Sigma-Aldrich (St. Louis, MO, USA). 10x Tris/Borate/EDTA (TBE) buffer, 30 % Acrylamide/Bis-acrylamide solution (29:1), Ladder Gene Rule 100 bp DNA and Quant-iT™ PicoGreen™ dsDNA Assay Kit were purchased from the Thermo Fisher Scientific Inc. (Waltham, MA, USA). The 96-well half-area plate (Cat. #29444-316) was purchased from VWR International (Secaucus, NJ, USA). RQ1 RNase-Free DNase-1 (Cat. #M6101) and the RQ1 DNase 10X Reaction Buffer (Cat. #M198A) were purchased from Promega Corporation (Madison, WI 53711-5399 USA). EnGen Lba Cas12a (Cpf1), NEBuffer 2.1 10X reaction buffer from New England BioLabs Inc. (Ipswich, MA, USA). Capillary Tubes (0.4mm I.D. Length 75mm) (Cat. #1-000-800) were purchased from Drummond Scientific Co. (Broomall, PA 19008). TwistAmp™ Basic RPA Kit (TABAS03KIT) was purchased from Twist Biosciences (San Francisco, CA, USA). The Fluorescent ruler was purchased from SP Bel-Art (South Wayne, NJ, USA). All DNA oligonucleotides, primers and crRNA constructs were purchased from Integrated DNA Technologies, Inc. (Coralville, IA, USA), **Table S1**.

Equipment. The Agilent Technologies Cary 60 UV-Vis was used to measure DNA concentration. The CORNING LSE™ Digital Dry Bath was used to anneal the duplexes and triplexes of the DNA. The Bio Rad Mini PROTEAN® Tetra Cell 552BR 182425 was used for gel electrophoresis. Bio Rad ChemiDoc™ MP Imaging System was used for gel imaging and fluorescence imaging. The Branson CPX2800H Digital heated Ultrasonic Cleaner was used to sonicate the Cyanuric acid solution. The Agilent BioTek Synergy H1 Multimode Plate Reader was used to measure the fluorescence intensity and kinetics over the time. The 2720 Thermal Cycler from Applied Biosystems was used for incubation and enzyme activation. A standard and a fluorescent ruler were used to measure the distance travelled by the hydrogel in the capilaty.

Identfying the truncated target and crRNA for *Salmonella*. To identify a targetable genomic region for *Salmonella Typhimurium*, genome sequences of AE006468, CP110657.1 were aligned using SnapGene software. Two highly conserved 24-base target regions with a TTTN PAM sequence were ($tDNA_{Typh3}$ and $tDNA_{Typh5}$) identified within the *invA* gene. The corresponding crRNAs was designed using the IDT website. To identify a targetable genomic region for *Salmonella Enteritidis*, genome sequences of AM933172, NC_011294, CP050723.1 were aligned. A conserved 24-base sequence target region with a TTTN PAM sequence was identified within the *invA* gene.[1,2] The corresponding crRNA was also designed using the IDT website.

Designing primers. RPA primers were designed through a comparative alignment of the *Salmonella Typhimurium* genomes AE006468 and CP110657.1. Of the four primer pairs previously tested, the combination of forward primer 2 (F2) and reverse primer 4 (R4) demonstrated the highest amplification efficiency and was selected for further experiments.[1] Primer selection adhered to the recommendations provided in the RPA kit protocol. Additionally,

a CRISPR RNA (crRNA) was designed using the IDT online tool to specifically target the amplified DNA sequence ($tDNA_{Typh3}$) and included the scaffold required for Cas12a recognition.

***S.* Typhimurium genome induced hydrogel dissasembly.** *S.* Typhimurium genome (catalog #700720D-5, batch #70029794) was purchased from American Type Culture Collection (ATCC; Manassas, VA, USA). The recombinase polymerase amplification reaction was performed using a commercial RPA kit according to the manufacturer's instruction (TwistAmp^{TM} Basic Kit). Where, 2.4 µL (10 µM) of forward primer, 2.4 µL (10 µL) of reverse primer, 29.5 µL of the Primer Free Rehydration buffer, 1.2 µL of target genome (1000 copy number), 12 µL of RDI water and 2.5 µL of magnesium acetate were mixed making total volume to 50 µL into a 1.5 mL microcentrifuge tube and then incubated at 37 °C for 30 min. This procedure was also performed without the *S. Typhimurium* genome for the control. The RPA reaction was performed with a final amount of 50 copies of the *S. Typhimurium genome* as detailed below.

The New England BioLabs Inc. Monarch PCR & DNA Cleanup Kit was used to clean the RPA product following the cleanup instructions in the kit. First, The RPA product was pipetted into a DNA cleanup column with 100 µL of DNA cleanup binding buffer. The column was then placed into collection tube and centrifuged for 1 min at 13,000 rpm. After discarding the flowthrough, 500 µL of DNA wash buffer was pipetted into the column and centrifuged for 1 min at 13,000 rpm. The column was then moved to a clean collection tube. 20 µL of DNA elution buffer was then pipetted into the column followed by centrifugation for 1 minute at 13,000 rpm. The column was then washed twice with 20 µL of 1X PBS buffer. The final elution was then diluted to a final volume of 100 µL using 1x PBS buffer which contains RPA amplicons products from 1000 copies (10copies/µL).

After the RPA product cleanup, the Cas12a-crRNA complex was prepared according to the proceduce described above. Then, 12.5 µL of the RPA products were immediately added to the 25 µL of freshly prepared Cas12a-crRNA complex making 50 µL of total volume with 12.5 µL of 1X reaction buffer and incubated at 37 °C for 20 minute (2.5copies/µL). After incubation, 20 µL (20 µLx2.5copies/µL = 50 copies) of the resulting solution was added to the DNA hydrogel (20µL) in 96-well plate followed by the addtion of 10 µL of 1X reaction buffer, to bring total volume of 50 µL. Fluorescence measurements were performed with an excitation wavelength 485 nm, emission at 520 nm, and a gain setting of 90.

The mobility assay was conducted using capillary tubes (0.4 mm width, 7.5 cm length) immersing vertically into a sterile half-area 96-well plate containing 50µL of the hydrogel mix solutions into different wells for 3 seconds and taken out. The distance travelled inside capillary tubes by the solutions were measured using ImageJ software.

Y-motif Preperation. Various buffer solutions with pH values ranging from 3 to 8 were prepared using the original 1xTAMg buffer (pH 6.5). Stock solutions of 5 mM for each of the three single-stranded DNA (ssDNA) strands (Y1-A15, Y2-A15, and Y3-A15) were initially prepared in distilled water and then diluted to obtain secondary stock solutions of 50 µM. A 10 µM Y-motif complex (Y1 + Y2 + Y3) was assembled at a 1:1:1 molar ratio by mixing 5 µL

of each ssDNA strand in 1×TAMg buffer (pH 6.5), followed by thermal annealing at 95 °C for 5 minutes and gradual cooling to room temperature.[3] Additionally, three partially assembled duplexes (Y1+Y2, Y1+Y3, and Y2+Y3) at 10 μM each were prepared using the same procedure at a 1:1 molar ratio. The concentrations of all stock and working solutions were confirmed by measuring absorbance at 260 nm using an Agilent Technologies Cary 60 UV-Vis spectrophotometer. Samples were incubated overnight in a refrigerator.

Gel Electrophoresis. Native PAGE gel electrophoresis was performed to confirm the formation of the Y-motif DNA, an assembly of the three ssDNA strands (Y1, Y2, and Y3), using 1×TAMg buffer (40 mM Tris, 7.6 mM $MgCl_2 \cdot 6H_2O$, 50 mM NaCl, pH 6.5) and 5 μL of 10 mg/mL ethidium bromide (EtBr) solution. The PAGE apparatus was thoroughly cleaned and assembled, ensuring a leak-proof setup by testing with water.

The gel was prepared using a resolving gel solution composed of 4.8 mL DI water, 2.7 mL 30% acrylamide/Bis mix, 2.5 mL resolving gel buffer, 100 μL of 10% APS, and 6 μL of TEMED. The stacking gel solution consisted of 3.4 mL DI water, 0.83 mL 30% acrylamide/Bis mix, 0.63 mL stacking gel buffer, 50 μL of 10% APS, and 5 μL of TEMED. The stacking gel was poured over the polymerized resolving gel, the comb was immediately inserted, and the gel was allowed to solidify for approximately 45 minutes.

After polymerization, the comb was carefully removed, and the electrophoresis chamber was filled with 1×TAMg buffer up to the appropriate level. Seven samples were prepared at 5 μM concentration from their respective stock solutions: three singlets (Y1, Y2, Y3), three dimers (Y1+Y2, Y1+Y3, Y2+Y3), and one triplex (Y1+Y2+Y3). Each sample was mixed with 2 μL of Bromophenol Blue loading dye. A low molecular weight DNA ladder (10 μL) was also loaded.

Each sample (10 μL) was loaded into the wells, the lid was placed on the tank, and the electrodes were connected. Electrophoresis was carried out at 100 V for 1 hour, or until the Bromophenol Blue dye front reached the bottom of the gel. After electrophoresis, the gel was washed with DI water for 5 minutes and stained with 5 μL of 10 mg/mL EtBr for 15 minutes. Imaging was performed using a Bio-Rad imaging system configured for EtBr-stained gels.

Preparation of CA-induced DNA hydrogel. Stock solutions of 200 μM Y-motif, 500 μM PolyA15 DNA, and 50 mM CA were prepared in 1×TAMg buffer (pH 6.5). These were mixed to obtain a final reaction mixture containing 50 μM Y-motif, 250 μM PolyA15, and 10 mM CA.[4] As a negative control, a separate mixture containing 50 μM Y-motif and 250 μM PolyA15 in the same buffer was prepared without CA. 2 μL of 1 % (w/v) stock solution of Bromophenol Blue dye was added to both the experimental and control mixtures and were thermally annealed at 37 °C for 1 hour and then incubated in the refrigerator overnight. Digital images of the samples were captured using a smartphone camera.

Mobility assay. A mobility assay was conducted at room temperature to evaluate both sample types: mixtures containing cyanuric acid and those without. Capillary tubes (75 mm in length, 0.4 mm diameter) were vertically immersed into 1.5 mL standard microcentrifuge tubes containing 200 μL of the hydrogel solution for 3 seconds to allow

capillary uptake. The tubes were then removed, and the extent of solution migration within each capillary was observed visually at room temperature and recorded with smartphone camera. Quantitative analysis of the distance traveled by the solution was performed using ImageJ software.

***DNase-I* responsive DNA hydrogel.** Stock solutions were prepared by mixing 200 µL of DNA hydrogel with 2 µL of PicoGreen dye (0.1/ µL DMSO). For the enzymatic digestion assay, a reaction mixture consisting of 40 µL of DNA hydrogel with PicoGreen, 5 µL of *DNase I* (1 U/µL), and 5 µL of RQ1 *DNase I* 10× Reaction Buffer was assembled and dispensed into a sterile half-area 96-well plate. As a negative control, a mixture of DNA hydrogel, PicoGreen, and PBS buffer (without *DNase I*) was prepared.

Fluorescence measurements were taken immediately using a Synergy BioTek H1 Microplate Reader. Kinetic data were collected at 2-minute intervals with an excitation wavelength of 485 nm, emission at 520 nm, and a gain setting of 100. Additionally, endpoint fluorescence spectra were recorded following the kinetic analysis.

The mobility assay was also conducted in triplicate using capillary tubes to compare the movement of hydrogel samples with and without *DNase I* treatment. Capillary tubes (75 mm length, 0.4 mm diameter) were vertically immersed in both 1.5 mL microcentrifuge tubes containing 100 µL of the respective hydrogel samples with and *without DNase I* for 3 seconds. The distance traveled by each sample within the capillaries was measured using ImageJ software. Both sample types, along with a fluorescent ruler, were imaged using a Bio-Rad imaging system, and additional photographs were captured using a smartphone camera.

Preparation of dsDNA target and nontarget strands. Stock solutions of 10 µM of dsDNAs were prepared for both target ($tDNA_{Typh3}$ + $cDNA_{Typh3}$) and non-target ($tDNA_{Ente}$ + $cDNA_{Ente}$) strands by hybridizing the complementary strands in a 1:1 ratio in 1× PBS buffer. These stock solutions were then diluted in a working cas buffer (10 mM Tris-HCl (pH 7.9), 50 mM NaCl, 10 mM $MgCl_2$, and 100 µg/mL BSA) to obtain secondary stock solutions at a concentration of 1 µM. Serial dilutions were prepared from these secondary stocks and used in subsequent experiments.

Cas12a-responsive DNA hydrogel. A 50 µL Cas12a-crRNA complex solution was prepared by mixing 0.5 µL of 100 µM Cas12a with 2.4 µL of 24.96 µM $crRNA_{Typhi3}$ in a 1:1.2 molar ratio, followed by the addition of 47 µL of Cas buffer (10 mM Tris-HCl, 50 mM NaCl, 10 mM $MgCl_2$, 100 µg/mL BSA, pH 7.9).[5] The mixture was incubated at 37 °C for 30 minutes in a PCR thermal cycler.

For the assay, 40 µL of DNA hydrogel containing PicoGreen was mixed with 3.75 µL of the 75 nM Cas12a-crRNA complex, 1 µL of 20 nM target $dsDNA_{Typhi3}$, and 5.25 µL of Cas buffer, then loaded into a sterile half-area 96-well plate. A negative control was prepared in the same manner, substituting the target with 1 µL of 20 nM non-target $dsDNA_{Ente}$. As a blank control, 50 µL of hydrogel containing PicoGreen alone was used.

Kinetic fluorescence measurements were initiated immediately and recorded over a 2-hour period. Endpoint fluorescence intensity was measured after the kinetic run. The mobility assay was also conducted using capillary tubes by vertically immersing in a sterile half-area 96-well plate containing 50 µL of the respective hydrogel solutions for 3 seconds.

The migration distance of each hydrogel sample was observed and quantitatively analyzed using ImageJ software. A concentration-dependent study was performed using 100 nM, 50 nM, 20 nM, and 0 nM of the $dsDNA_{Typhi3}$ target. Both the PicoGreen fluorescence assay and the mobility assay were conducted following the procedures above.

Selectivity. The Cas12a–crRNA complex was prepared using either either $crRNA_{Typhi}$ or $crRNA_{Ente}$ in Cas buffer (10 mM Tris-HCl, 50 mM NaCl, 10 mM $MgCl_2$, 100 µg/mL BSA, pH 7.9) and incubated at 37 °C for 30 minutes. The reaction mixture contained 30 µL of DNA hydrogel, 75 nM final concentration of the Cas12a complex, and 20 nM of the corresponding target DNA (either *S. Typhi* or *S. Ente*) in a total volume of 40 µL with Cas buffer in 600 µL microcentrifuge tubes. The reaction mixtures were incubated at room temperature for 1 hour. Each Cas12a complex was tested against either $dsDNA_{Typhi3}$ or $dsDNA_{Ente}$ For the blank control, no target DNA was added to the Cas12a and DNA hydrogel mixture. Capillary tubes (75 mm in length, 0.4 mm in diameter) were vertically immersed into the reaction mixtures for 3 seconds.

Payload Encapsulation and release of with CRISPR Cas12a. A mixture of 200 µL Y-motif and 500 µL PolyA15 was prepared, resulting in final concentrations of 50 µM Y-motif and 200 µM PolyA15 in 1x TAMg buffer (pH 6.5). To this mixture, 20 µL of various small molecules, nanoparticles, or a protein (as listed in **Table S2**) were added individually.[3] Each reaction mixture was incubated at 37 °C for 1 hour. Subsequently, 40 µL of a 50 mM cyanuric acid stock solution was added along with working buffer to bring the total volume to 200 µL, forming the hydrogel encapsulating the payloads. These samples were then heated for an additional hour at 37 °C and incubated overnight at 4 °C. Encapsulation was confirmed by capturing digital images with a smartphone and fluorescence images using a Bio-Rad imaging system programmed for the appropriate excitation/emission wavelengths, as detailed in **Table S2**. Nanoparticle conjugates were prepared following protocols described in our previous publications.[6,7]

To evaluate release of the encapsulated components, 30 µL stock solutions of 1 µM Cas12a-$crRNA_{Typhi5}$ complex, 1 µM target $dsDNA_{Typhi5}$, and 1 µM non-target $dsDNA_{Ente}$ were prepared. Each encapsulated hydrogel sample was divided into two batches. One batch was treated with the Cas12a-$crRNA_{Typhi5}$ complex and the target $dsDNA_{Typhi5}$, while the other was treated with the same complex but with non-target $dsDNA_{Ente}$. The final concentrations in each treated hydrogel were adjusted to 75 nM Cas12a-crRNA and 20 nM dsDNA. Samples were incubated at 4 °C for 2 hours. The final volume of each DNA hydrogel used for the mobility assay was calculated to be approximately 200 µL.

The mobility assay was conducted by vertically immersing capillary tubes into 2.0 mL microcentrifuge tubes containing 200 µL of each hydrogel sample for 3 seconds. To demonstrate fluorescence-based monitoring of mobillty, fluorescence

images were taken using the Bio-Rad imaging system, and the distances traveled by the hydrogel within the capillaries were quantified using ImageJ software.

Statistical Analysis. The experiments were performed in triplicate and the error bars were calculated by standard deviation (SD). Data are represented as Mean ± SD.

Table S1. List of oligonucleotide sequences used. For crRNA, the recognition region is in bold and underlined. For truncated *Salmonella* targets, target recognition site is in bold and underlined, with PAM highlighted in yellow.

**Label**	**Sequence**
Y1-A15	5'- AAA AAA AAA AAA AAA TGG ATC CGC ATG ACA TTC GCC GTA AG -3'
Y2-A15	5'- AAA AAA AAA AAA AAA CTT ACG GCG AAT GAC CGA ATC AGC CT -3'
Y3-A15	5'- AAA AAA AAA AAA AAA AGG CTG ATT CGG TTC ATG CGG ATC CA G -3'
A15	5'- AAA AAA AAA AAA AAA-3'
crRNA$_{Typh3}$	5'- UAA UUU CUA CUA AGU GUA GAU **UCG AGA UCG CCA AUC AGU CCU** -3'
crRNA $_{Typh5}$	5'- UAA UUU CUA CUA AGU GUA GAU **CAG UAC GCU UCG CCG UUC GCG** -3'
tDNA$_{Typh3}$ (truncated *S*. Typhimurium target i.e., ssDNA$_{Typh3}$)	5'- TCG TCG TTA **GGA CTG ATT GGC GAT CTC GAT AAA** GTC TCT ACA GAG ACC GT -3'
cDNA$_{Typh3}$ (complementary to tDNA$_{Typh3}$)	5'- ACG GTC TCT GTA GAG AC**T TTA TCG AGA TCG CCA ATC AGT CC**T AAC GAC GA -3'
tDNA$_{Ente}$ (truncated *S*. Enteritidis target i.e., ssDNA$_{Ente}$)	5'- GGT ATC TGC T**GA AGT TGA GGA TGT TAT TCG CAA A**GG GAT CCG TCA GAC CT -3'
cDNA$_{Ente}$ (complementary to tDNA$_{Ente}$)	5'- AGG TCT GAC GGA TCC C**TT TGC GAA TAA CAT CCT CAA CTT C**AG CAG ATA CC -3'
tDNA$_{Typh5}$ (truncated *S*. Typhimurium target i.e., ssDNA$_{Typh5}$)	5'GAT GCG GAT GCC GCG **CGC GAA CGG CGA AGC GTA CTG GAA A**GG GAA AGC CA -3'
cDNA$_{Typh5}$ (complementary to tDNA$_{Typh5}$)	5'- TGG CTT TCC C**TT TCC AGT ACG CTT CGC CGT TCG CG**C GCG GCA TCC GCA TC -3'
2F (forward primer 2)	5'- CTC CGC AAG TTG AGC TTT TTC CAG ATC TTC AC -3'
4R (reverse primer 4)	5'- CAG TAT TGA GGA AAA AGA AGG GTC GTC GTT AG -3'

Table S2. List of payloads used for the encapsulation and release studies. Excitation wavelengths, stock concentrations, and the imaging channels (blots) used on the Bio-Rad imaging system are provided.

No.	Payload	Excitation	Stock	Blot used
1	TAMRA	555 nm	~ 60 ng/mL (500 μM)	(Fluorescein Gels 590/110, UV Trans, Exposure – 0.1 s)
2	Doxorubicin (Dox)	500 nm	~ 54 ng/mL (1mM)	(Fluorescein Gels 590/110, UV Trans, Exposure – 0.1 s)
3	MN_{Dox}	500 nm	3.0 μM	(Fluorescein Gels 590/110, UV Trans, Exposure – 0.1 s)
4	$MN_{Cy5.5}$	675 nm	3.5 μM	(Cy5.5 Blot 715/30, Far Red Epi, Exposure – 0.1 s)
5	Red Avidin	590 nm	1.8 mg/mL	(Rhodamine 602/50 – Green Epi, Exposure – 0.1 s)

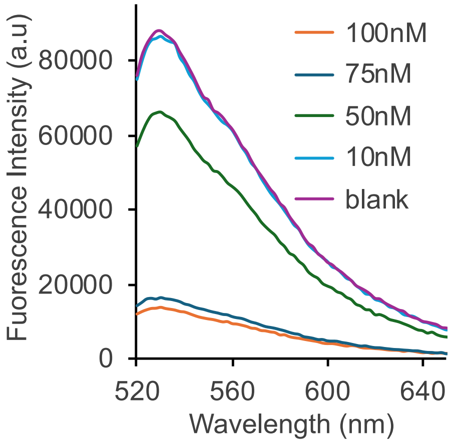

Figure S1. Fluorescence spectra of PicoGreen-stained DNA hydrogels treated with different concentrations of Cas12a–crRNA$_{Typh3}$ complex and 20 nM dsDNA$_{Typh3}$.

REFERENCES:

(1) Kachwala, M. J.; Hamdard, F.; Cicek, D.; Dagci, H.; Smith, C. W.; Kalla, N.; Yigit, M. V. Universal CRISPR-Cas12a and Toehold RNA Cascade Reaction on Paper Substrate for Visual Salmonella Genome Detection. *Adv. Healthc. Mater.* **2024**, *13* (22), 2400508. https://doi.org/https://doi.org/10.1002/adhm.202400508.

(2) Hanson, E.; Kalla, N.; Tharu, R. J.; Demir, M. M.; Tok, B. H.; Canbaz, M. A.; Yigit, M. V. CRISPR-Responsive Reprogrammable Label-Free Fluorescent Nanoclusters for ML-Assisted Pathogenic Genome Detection on Solid Substrates. *Small* **2025**. https://doi.org/10.1002/smll.202500784.

(3) He, M.; Nandu, N.; Uyar, T. B.; Royzen, M.; Yigit, M. V. Small Molecule-Induced DNA Hydrogel with Encapsulation and Release Properties. *Chem. Commun.* **2020**, *56* (53). https://doi.org/10.1039/d0cc03439h.

(4) Avakyan, N.; Greschner, A. A.; Aldaye, F.; Serpell, C. J.; Toader, V.; Petitjean, A.; Sleiman, H. F. Reprogramming the Assembly of Unmodified DNA with a Small Molecule. *Nat. Chem.* **2016**, *8* (4). https://doi.org/10.1038/nchem.2451.

(5) Smith, C. W.; Kachwala, M. J.; Nandu, N.; Yigit, M. V. Recognition of DNA Target Formulations by CRISPR-Cas12a Using a DsDNA Reporter. *ACS Synt. Bio.* **2021**, *10* (7). https://doi.org/10.1021/acssynbio.1c00204.

(6) Robertson, N. M.; Yang, Y.; Khan, I.; Lamantia, V. E.; Royzen, M.; Yigit, M. V. Single-Trigger Dual-Responsive Nanoparticles for Controllable and Sequential Prodrug Activation. *Nanoscale* **2017**, *9* (28). https://doi.org/10.1039/c7nr04138a.

(7) Uyar, T. B.; Wu, K.; He, M.; Khan, I.; Royzen, M.; Yigit, M. V. Switchable Fluorescence of Doxorubicin for Label-Free Imaging of Bioorthogonal Drug Release. *ChemMedChem* **2020**, *15* (11). https://doi.org/10.1002/cmdc.202000065.
